# The first Chinese national standards for SARS-CoV-2 neutralizing antibody

**DOI:** 10.1016/j.vaccine.2021.05.047

**Published:** 2021-06-23

**Authors:** Lidong Guan, Yuanling Yu, Xiaohong Wu, Jianhui Nie, Jun Zhang, Zejun Wang, Na Li, Rui Shi, Hui Zhao, Hongbo Chen, Chunxia Luo, Yaling Hu, Youchun Wang, Weijin Huang, Miao Xu, Jifeng Hou

**Affiliations:** a Institute for Biological Product Control, National Institutes for Food and Drug Control (NIFDC) and WHO Collaborating Center for Standardization and Evaluation of Biologicals, Beijing, China; b Beijing Institute of Biotechnology, Academy of Military Medical Science, Beijing, China; c Wuhan Institute of Biological Products Co., Ltd., Wuhan, Hubei, China; d Beijing Institute of Biological Products Co., Ltd., Beijing, China; e Institute of Microbiology, Chinese Academy of Sciences, Beijing, China; f State Key Laboratory of Pathogen and Biosecurity, Beijing Institute of Microbiology and Epidemiology, Beijing, China; g Institute of Medical Biology, Chinese Academy of Medical Sciences, Kunming, Yunnan, China; h Sinocelltech Ltd., Beijing, China; i Sinovac Biotech Ltd., Beijing, China

**Keywords:** SARS-CoV-2, COVID-19, Neutralizing antibody, Standard, Neutralization assay

## Abstract

In order to meet the domestic urgent needs of evaluating the immunogenicity of vaccines and the potency testing of therapeutic antibody products against coronavirus disease 2019 (COVID-19), the first Chinese national standards for SARS-CoV-2 neutralizing antibody were established. The potency and stability of the candidate standards were determined by neutralization assay and accelerated degradation study. The stability studies showed that the standards were stable in the short-term. The collaborative study showed that the candidate standards could reduce the variations in neutralization titers between labs and thus improve comparability of neutralizing antibody measurements. Sample 22 has been approved by the Biological Product Reference Standards Sub-Committee of the National Drug Reference Standards Committee as the first Chinese National Standard for Severe Acute Respiratory Syndrome Coronavirus-2 (SARS-CoV-2) neutralizing antibody, with an assigned potency of 1,000 units per milliliter (U/ml). This standard will contribute to the standardized assessment of the quality and efficacy of vaccines and therapeutics for COVID-19 in China.

## 1 Introduction

On December 31, 2019, unexplained pneumonia (later designated as coronavirus disease 2019, COVID-19) broke out in Wuhan, China [1]. The causative agent of COVID-19, Severe Acute Respiratory Syndrome Coronavirus-2 (SARS-CoV-2), is highly pathogenic and infectious, and can be transmitted through contact, droplets, aerosols, and fecal-orally [2]. COVID-19 has now become a global pandemic, threatening the public health and economic development of countries across the world. As of October 28, 2020, more than 43 million cases of COVID-19 have been diagnosed and over one million people have died of the disease worldwide. Thus, vaccines and therapeutics for COVID-19 are urgently needed. Currently, the World Health Organization (WHO) and world governments are working together to accelerate the development of prophylactics and therapeutics to counter this pandemic [3], [4]. However, different from other respiratory diseases caused by coronaviruses, such as Severe Acute Respiratory Syndrome (SARS) and Middle East Respiratory Syndrome (MERS), COVID-19 is a brand-new disease and there are currently no wholly effective prevention and treatment countermeasures. Preventive vaccines and therapeutic neutralizing antibodies are important means for the prevention and control of COVID-19, and are the key research objectives of institutions and pharmaceutical companies looking to help control the pandemic [5], [6], [7], [8], [9], [10], [11], [12]. Neutralizing antibodies can inactivate the SARS-CoV-2 virus, presumably by intercepting the virus before it attaches to the surface of the target cell. The level of neutralizing antibodies is a key indicator for assessing the immunogenicity of vaccines and the potency of therapeutics, and it must be determined accurately.

By October 29, 2020, 13 vaccines and one antibody product against COVID-19 had entered clinical phases in China [13]. Yet, there are no standardized methods to measure the neutralizing antibody against SARS-CoV-2. These relevant differences in test protocols have resulted in the incomparability of assay data between labs and products. Standardized neutralization assay methods depend on standards for neutralizing antibodies. In addition, standard is an indispensable tool for controlling the quality of medicines in drug inspection. The standards for SARS-CoV-2 neutralizing antibody are thus urgently needed. In this study, the establishment of the first national standards for neutralizing antibodies to SARS-CoV-2 are described. The standards were produced from plasma samples from convalescent COVID-19 patients in China. This collaborative study was organized by the National Institutes for Food and Drug Control (NIFDC). Eleven labs, including national research labs, national control labs, and manufacturers of vaccines and antibody products engaged in this study. The establishment of these national standards aimed to standardize the methods for neutralization assays, ensure the accuracy and comparability of neutralizing antibody titers between different labs and products, and effectively control the quality of vaccines and therapeutics for COVID-19.

## 2 Materials and methods

### 2.1 Ethics statement

Two plasma samples from COVID-19 convalescent patients, sample 22, sample 77 were generously provided by Boya Bio-pharmaceutical Group Co., Ltd. and Sinopharm Wuhan Plasma-derived Biotherapies Co., Ltd.. Written informed consents were obtained from all the volunteers.

### 2.2 Preparation of candidate standards for SARS-CoV-2 neutralizing antibody

Two plasma samples from convalescent patients were first tested for hepatitis B virus deoxyribonucleic acid (HBV DNA), hepatitis C virus ribonucleic acid (HCV RNA), human immunodeficiency virus (HIV) RNA, and SARS-CoV-2 RNA, as well as the corresponding Hepatitis B surface antigen (HBsAg), HIV-1 / HIV-2 antibody, HCV antibody, and syphilis antibody. Meanwhile, the plasma was inactivated at 56 °C for 30 min.

The neutralizing antibody titer of convalescent COVID-19 patient plasma was detected by a pseudo virus neutralization assay as described previously [14], [15]. In brief, the serial dilutions of the test samples (six dilutions in a 3-fold step-wise manner) were incubated with pseudo virus for one hour at 37 °C, together with the virus control and cell control wells. Then, freshly trypsinized Huh-7 cells (2 × 10^4^ cells) were added to each well. Following 24 h of incubation in a 5% carbon dioxide (CO_2_) environment at 37 °C, the relative light unit (RLU) was detected according to the instruction manual provided by PerkinElmer (Waltham, MA). The neutralization titers were defined as 50% maximal inhibitory dilutions and calculated with the Reed-Muench method. Inhibition percentage was calculated as the following: [1− (RLU in test samples − RLU in cell controls) / (RLU in virus controls − RLU in cell controls)] × 100%.

The plasma with higher titer neutralizing antibodies was selected to prepare candidate standards for this study. After being inactivated, the plasma was placed in the biosafety cabinet until it reached room temperature, and then kept at 4 °C overnight. The next day, the plasma samples were centrifuged at 5,000 revolutions per minute (rpm) for 15 min. The supernatant was separated and packed into 0.5 ml (ml) screw cap tubes, with each tube containing 100 Âµl (ul), which was then sealed and stored at **−** 30 °C.

### 2.3 Design of collaborative calibration samples

A total of five samples were provided to participants:

• Sample 22 was convalescent COVID-19 patient plasma provided by Boya Bio-pharmaceutical Group Co., Ltd.
• Sample 77 was convalescent COVID-19 patient plasma provided by Sinopharm Wuhan Plasma-derived Biotherapies Co., Ltd.
• Sample 44 was prepared by mixing SPF rabbit antiserum (provided by Prof. Changgui Li, NIFDC; antigen: recombinant SARS-CoV-2 spike 1 protein) with plasma from healthy donors.
• Sample 99 was prepared by mixing rabbit antiserum (provided by Prof. Changgui Li, NIFDC; antigen: SARS-CoV-2 spike receptor-binding domain (RBD) protein) with plasma from healthy donors.
• Sample 55 was human plasma, which was derived from a large mixture of plasma from healthy donors.

### 2.4 Participants

The following eleven labs engaged in the collaborative study: the Institute of Microbiology, Chinese Academy of Sciences; the Institute of Medical Biology, Chinese Academy of Medical Sciences ; the Sinocelltech Ltd.; the Wuhan Institute of Biological Products Co., Ltd.; the Institute of Biotechnology, Academy of Military Medical Sciences; the Institute of Microbiology and Epidemiology, Academy of Military Medical Sciences; the Sinovac Biotech Ltd.; the Beijing Institute of Biological Products Co., Ltd.; the Division of Arbovirus Vaccines, National Institutes for Food and Drug Control (NIFDC); the Division of HIV/acquired immunodeficiency syndrome (AIDS) and Sexually Transmitted Virus Vaccines, NIFDC ; and the Division of Blood Products, NIFDC. All labs are assigned code numbers and not reflected in the order presented in Table 1.

### 2.5 Collaborative calibration study

The NIFDC organized this collaborative study. The eleven labs mentioned above participated in the study. Live virus- and pseudo virus-based neutralization assays were used to detect the neutralization titer against SARS-CoV-2 (Table 1). The pseudo virus neutralization assay was performed according to the method established by Nie *et al.* [15]. The live virus neutralization assay was conducted using participant's established methods. For each sample, at least three independent assays were performed. In each assay, a fresh tube of sample was used and more than six dilutions for each sample was required.

**Table 1:** Summary of Neutralization Assay Methods in the Collaborative Calibration Labs.

**Lab**	**Introduction of Method**	**Detection of Result**
WS1,ZX,SZ,ZA,ZC,ZW,JW,JS1,BS,ZY1	Virus:SARS-CoV-2 pseudo virus,Cell:Huh-7	Relative light unit
BK	Virus:SARS-CoV-2, CZ01 (non-vaccine strain),Cell:Vero	Cytopathic effect
JS2	Virus:SARS-CoV-2, serial No. GWHABKZ01000000 (non-vaccine strain),Cell:Vero-E6	Cytopathic effect, OD_570_ after staining with crystal violet
ZY2	Virus:SARS-CoV-2, KMS2 (non-vaccine strain),Cell:Vero	Cytopathic effect
WS2	Virus:SARS-CoV-2, WIV04 (vaccine strain),Cell:Vero	Plaques
WS3	Virus:SARS-CoV-2, WIV04 (vaccine strain),Cell:Vero	Cytopathic effect

*Abbreviations:* OD, optical density; SARS-CoV-2: Severe Acute Respiratory Coronavirus-2

### 2.6 Stability study

Due to the inadequate time and plasma with high titer neutralizing antibody against SARS-CoV-2, we did not conduct long-term stability studies on Sample 22, and only did partial temperature acceleration tests in this study. Sample 22 was separately stored at − 30 °C, 4 °C, and 25 °C, and the titers of neutralizing antibody were determined on days 7, 11, 18, 27, and 33 by pseudo virus neutralization assay.

### 2.7 Neutralizing activity of Sample 22 against variants of concern

The pseudotyped viruses corresponding to the SARS-CoV-2 variants B.1.1.7, B.1.351 and P.1 were constructed using the methods reported in our previous papers [14], [15]. The pseudo-virus neutralization assay was performed according to the method established by Nie *et al.* [15]. The sample ED_50_ (median effective dilution) was calculated using the Reed-Muench method [15].

### 2.8 Statistical analysis

The neutralization titers in the collaborative study samples were assessed by neutralization assays based on pseudo virus or live virus. For the pseudo virus-based neutralization assay, the neutralization titer was calculated by Reed-Muench method based on the results of the neutralization inhibition rate [15]. For the live virus-based neutralization assay, one lab detected the virus by plague staining, one lab detected absorbance after staining with crystal violet, and three labs detected cytopathic effect (CPE) (Table 1). The sample neutralization titer was calculated using the Reed-Muench method. The Reed-Muench method has been widely used in China to calculate the neutralization titers of antibody products and vaccine-elicited sera. The geometric mean (GM) of at least three independent assays performed in order to calculate the laboratory mean of each sample. Overall mean potencies were calculated as the geometric means of the laboratory means. The variations in the neutralizing titer between assays within labs and between labs were assessed using geometric coefficients of variation (GCV), with the formula GCV% = (e ^s-1) × 100% where s is the standard deviation of the lnN transformed results. Before statistical analysis, the natural logarithm of potencies was transformed to fit normal distribution [16].

Minitab 17 was used for statistical analysis. GraphPad Prism 8 (GraphPad Software, Inc., San Diego, CA, USA) was used for correlation analysis. Based on the results of the pseudo virus neutralization assay, parallel-line analysis (PLA 3.0 software) was used to trace the first Chinese national standard for SARS-CoV-2 neutralizing antibody (Sample 22) to the WHO IS (Sample G).

## 3 Results

### 3.1 Screening of candidate standards for SARS-CoV-2 neutralizing antibodies

To screen plasma with high neutralizing potency, we detected the neutralizing antibody against SARS-CoV-2 in two plasma samples from Boya Bio-pharmaceutical Group Co., Ltd. and Sinopharm Wuhan Plasma-derived Biotherapies Co., Ltd. The geometric mean values of the neutralizing antibody titers in the two samples were 1307 and 136, respectively. The BOYA sample (No. 22), with a higher neutralizing antibody titer, was selected as the candidate national standard for SARS-CoV-2 neutralization antibodies.

### 3.2 Results of collaborative calibration

Ten of the eleven labs returned the results of the pseudo virus neutralization assay, which are listed in the Table S1. Four labs returned live virus neutralization assay results, which are shown in the Table S2. Among them, both Lab JS and Lab ZY returned one set of pseudo virus neutralization assay results and one set of live virus neutralization assay results. Lab WS returned one set of pseudo virus neutralization assay results and two sets of results from two different live virus neutralization assays. Sample 55 was a negative control; the results of different labs were consistent and will not be analyzed in the future study.

### 3.3 The geometric mean potencies for samples in the pseudo virus neutralization assay

**Fig. 1:**
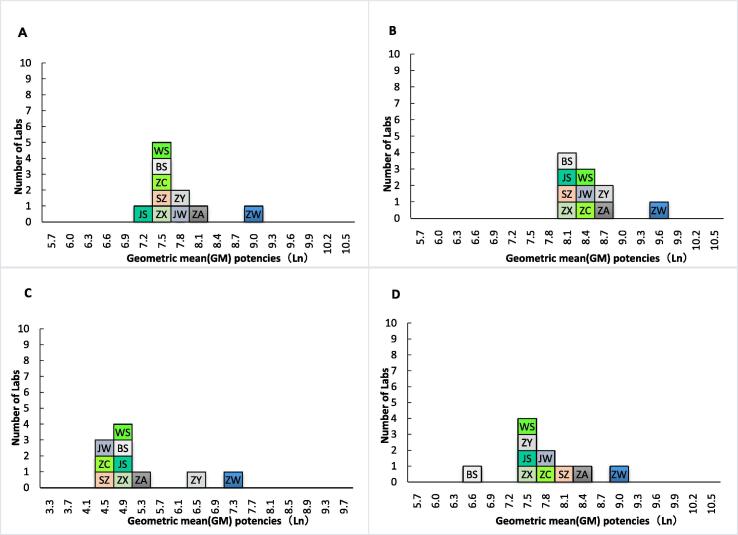
Laboratory geometric mean potencies for Samples 22 (A), 44 (B), 77 (C), and 99 (D).

The geometric mean potencies for samples in the pseudo virus neutralization assay are shown in Table 2 and Fig. 1. Because Sample 44 in Labs ZW and ZA had data for only one valid assay, the intra-lab (between replicate assay) variability was not calculated. Except for Sample 77 in Lab ZW (GCV of 324%), the intra-lab coefficient of variation of each sample was less than 100%. As shown in Table 2, Lab ZW had significantly higher potency estimates for each sample than that of other labs. The overall geometric mean estimates for Samples 22, 44, 77, and 99 were 1,938, 3,973, 162, and 2,064, respectively, the pooled GCV between labs for Samples 22, 44, 77, and 99 were 63%, 56%, 172%, and 88%, respectively (Table 2). If Sample 22 was set as the standard and assigned a potency of 1,000 units per milliliter (U/ml), the high potency estimates in Lab ZW was corrected, and the overall GCV between labs for Samples 44, 77, and 99 decreased to 23%, 106%, and 48%, respectively (Table 3).

**Table 2:** Geometric Mean Potencies for Samples in the Pseudo virus Neutralization Assay.

**Lab**		**Sample**										
		***22***			***44***		***77***			***99***		
	**N**	**GM**	**GCV%**	**N**	**GM**	**GCV%**	**N**	**GM**	**GCV%**	**N**	**GM**	**GCV%**
WS1	8	1613	*21*	8	4306	*15*	9	113	*39*	9	1711	*19*
ZX	4	1460	*19*	4	2491	*12*	4	102	*33*	4	1474	*7*
SZ	3	1455	*7*	3	3045	*27*	3	81	*79*	3	2978	*49*
ZA	3	3271	*46*	1	5076	*NC*	3	163	*12*	3	3509	*16*
ZC	3	1498	*27*	3	3740	*12*	3	64	*61*	3	2284	*87*
ZW	3	6117	*44*	1	11,603	*NC*	3	1478	*324*	2	7528	*41*
JW	3	2185	*59*	3	3444	*56*	4	87	*27*	4	1864	*38*
JS1	4	1268	*14*	4	2942	*14*	4	127	*10*	4	1713	*18*
BS	3	1431	*20*	3	2747	*10*	3	120	*44*	3	717	*34*
ZY1	3	1835	*58*	3	4898	*27*	3	638	*85*	3	1352	*54*
Overall GM		1938			3973		162			2064		
Overall GCV%		63			56		172			88		
CI (95%)		1367–2748			2886–5470		79–330			1314–3243		
Overall GM excluding ZW		1706			3527		126			1788		
Overall GCV% excluding ZW		34			29		95			59		
CI (95%) excluding ZW		1385–2101			2938–4234		79–203			1282–2493		

*Abbreviations:* N, number of assays; NC, not calculated; GM, geometric mean; GCV, geometric coefficient of variation; CI, confidence interval.

**Table 3:** Potencies Expressed Relative to Sample 22 in the Pseudo Virus Neutralization Assay (U/ml).

**Lab**	**Sample**			
	***22***	***44***	***77***	***99***
WS1	1000	2347	62	932
ZX	1000	1706	70	1009
SZ	1000	2093	56	2047
ZA	1000	1552	50	1073
ZC	1000	2497	43	1525
ZW	1000	1897	242	1231
JW	1000	1576	40	853
JS1	1000	2319	100	1350
BS	1000	1920	84	501
ZY1	1000	2669	348	737
Overall GM relative to 22		2050	83	1065
Overall GCV% relative to 22		23	106	48
Overall GM relative to 22 excluding ZW		2068	74	1048
Overall GCV% relative to 22 excluding ZW		24	92	51

*Abbreviations:* GM, geometric mean; GCV, geometric coefficient of variation.

### 3.4 The geometric mean potencies for samples in the live virus neutralization assay

The geometric mean estimates for samples in the live virus-based neutralization assay are listed in Table 4. Lab WS returned two sets of assay data, which were based on the plaque reduction neutralization test (PRNT) and CPE. The geometric mean potencies based on these two methods were quite different. The geometric mean potencies of Lab ZY and Lab WS based on the CPE were relatively close, but were quite different from the potencies of Lab BK, which also used the CPE method. Lab JS's virus challenge strain, cells, and results-detection methods were different from the other three labs, and its geometric mean potencies were lower than those of other labs. Since Sample 77 in Lab JS had no valid assay data, the geometric mean potency and intra-lab variability was not calculated. As shown in Table 4, the intra-lab GCVs ranged from 0 to 92% depending on the samples, which were significantly lower than the GCVs between labs. The GCV between labs for Samples 22, 44, 77, and 99 was 360%, 129%, 266%, and 146%, respectively. If Sample 22 was set as the standard and assigned a potency of 1,000 U/ml, the GCV between the labs for Samples 44, 77, and 99 decreased to 107%, 18%, and 90%, respectively (Table 5).

**Table 4:** Geometric Mean Potencies for Samples in the Live Virus Neutralization Assay.

**Lab**		**Sample**										
		***22***			***44***			***77***		***99***		
	N	GM	GCV%	N	GM	GCV%	N	GM	GCV%	N	GM	GCV%
BK	3	1219	49	3	1691	18	3	161	49	3	1536	0
JS2	5	33	48	5	282	21	5	NC	NC	5	201	43
ZY2	3	121	49	3	645	49	3	15	74	3	369	42
WS2	3	1063	13	3	2308	12	3	183	92	3	1463	39
WS3	9	200	35	9	914	41	9	24	38	9	426	53
Overall GM		252			917			57		589		
Overall GCV%		360			129			266		146		
CI (95%)		38–1679			328–2563			7–446		193–1797		

*Abbreviations:* N, number of assays; NC, not calculated; GM, geometric mean; GCV, geometric coefficient of variation; CI, confidence interval.

**Table 5:** Potencies Expressed Relative to Sample 22 in the Live Virus Neutralization Assay (U/ml).

**Lab**	**Sample**			
	***22***	***44***	***77***	***99***
BK	1000	1387	132	1260
JS	1000	8545	NC	6091
ZY	1000	5333	120	3053
WS	1000	2171	172	1377
WS	1000	4579	121	2132
Overall GM relative to 22		3628	135	2331
Overall GCV %		107	18	90

*Abbreviations:* NC, not calculated; GM, geometric mean; GCV, geometric coefficient of variation.

### 3.5 Correlation between live SARS-CoV-2- and pseudo virus-based neutralization assays

**Fig. 2:**
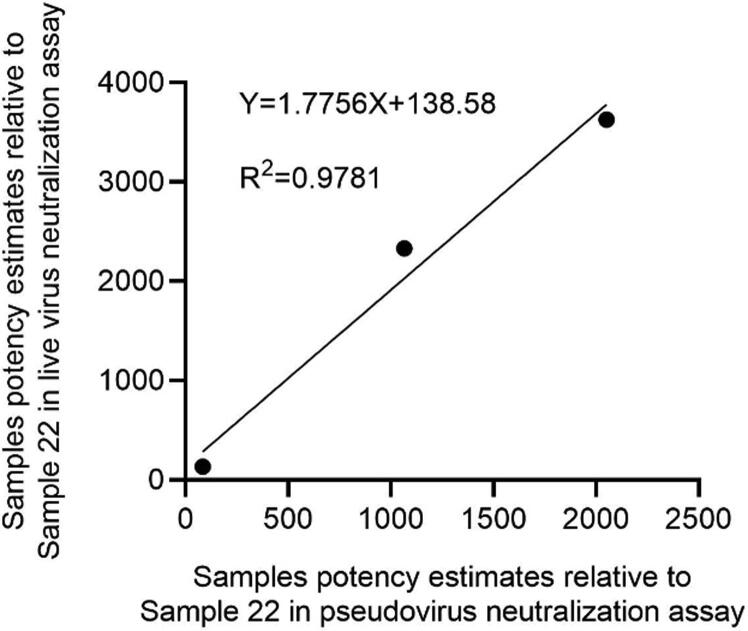
Comparison the samples potency estimates relative to Sample 22 obtained from the pseudo virus neutralization assay with the live virus neutralization assay.

It can be seen from the previous assay data that due to the differences in assay method, virus challenge strain, and cells between the live virus- and pseudo virus-based neutralization assays, the neutralizing antibody titers of the same sample were quite different (Table 2 and Table 4). We then compared the samples potency estimates relative to Sample 22 obtained from the pseudo virus neutralization assay with the live virus neutralization assay. Although the samples’ potencies against pseudo virus were lower than those against live SARS-CoV-2 after standardization, there was a high correlation between the two assays (R^2^ = 0.9781) (Fig. 2).

### 3.6 Stability study

Our previous results showed that the geometric mean of the neutralizing antibody titer of Sample 22 was 1,460 (95% confidence interval 1,112–1,918). The results of this accelerated test showed that within one month, whether Sample 22 was stored at − 30 °C, 4 °C, or 25 °C, the changes in the titer of the neutralizing antibodies were within the 95% confidence interval. There was no significant loss of neutralization activity at 4 °C and 25 °C relative to the − 30 °C baseline for Sample 22 (Table 6). These results indicated that Sample 22 has good short-time stability. Samples will be tested yearly to assess long-term stability.

**Table 6:** Accelerated degradation study of Sample 22.

**Time**	**Temperature**			**Potencies Relative to − 30 °C Baseline**	
	**−30 °C**	**4 °C**	**25 °C**	**4 °C**	**25 °C**
Day 7	1249	1443	1133	1.16	0.91
Day 11	1444	1586	1370	1.10	0.95
Day 18	991	1557	1457	1.57	1.47
Day 27	1190	1391	1762	1.17	1.48
Day 33	1344	1093	1262	0.81	0.94
Geometric mean	1234	1402	1381	1.14	1.12

## 4 Discussion

Due to the high pathogenicity and infectivity of SARS-CoV-2, COVID-19 has become a global pandemic, causing tremendous devastation to the world. Currently, there are no approved effective anti-viral products or vaccines against SARS-CoV-2. Accurate determination of neutralizing antibodies against SARS-CoV-2 is critical for evaluating the immunogenicity of vaccines, assessing the potency of antibody products, and establishing quality control standards for vaccines and antibody products. Both the live virus-based neutralization assay, such as PRNT and CPE, and pseudo virus-based neutralization assays were used to detect neutralization antibody titers [5], [7], [17], [18], [19]. Because different labs use different neutralization assay methods, or the neutralization assay methods are the same but the virus challenge strains, cells, and detection methods are different, the assay data between different labs and various products are not comparable. In order to standardize the virus neutralization method and ensure the accuracy and comparability of assay data, this study aimed to develop national standards for SARS-CoV-2 neutralizing antibodies.

To assess the suitability of candidate national standard virus neutralization assays, the collaborative calibration of five samples was conducted in eleven labs across China. In this collaborative study, although the method of pseudo virus neutralization assay was unified, there were differences in the testing conditions, cell status, and personal operations in various labs, which affected the consistency of results between labs (Table 2). In the live virus neutralization assay, due to the differences in virus strains and cells, as well as methods of observing the results, the GCVs across labs were higher (Table 4). However, once the values were expressed as relative potencies against the candidate standards, the variations in neutralizing titer between labs were significantly reduced in both live virus- and pseudo virus-based neutralization assays (Table 3 and Table 5). These results suggest that the application of standards could reduce discrepancies and improve comparability between assays performed in different labs. Although the samples’ potencies against pseudo virus were lower than those against live SARS-CoV-2 after standardization, there was a high correlation between the two assays (Fig. 2). Due to the limited number of samples in this test, further verification is needed in the future study. Consistent with our results, Li *et al.* reported a strong correlation between the neutralization antibody titers generated by pseudo virus and live virus systems [20]. Additionally, due to time constraints, we only did a short-term stability study, which showed that the candidate standard sample was stable in the short term (Table 6). However, more stability data are needed to establish a long-term stability profile. Taken together, Sample 22 is recommended as the national standard for the SARS-CoV-2 neutralizing antibody. The establishment of this national standard would ensure the accuracy and comparability of neutralizing antibody detection and facilitate the assessment of vaccine immunogenicity and antibody products potency.

## 5 Conclusions

Sample 22 was approved by the Biological Product Reference Standards Sub-Committee of the National Drug Reference Standards Committee as the first Chinese National Standard for SARS-CoV-2 neutralizing antibody in September 2020, with an assigned potency of 1,000 U/ml (Lot: 280034–202001). The WHO IS for anti-SARS-CoV-2 antibody was established in December 2020, with an arbitrary assigned unitage of 250 IU/ampule for neutralizing activity [21]. The first Chinese National Standard for SARS-CoV-2 neutralizing antibody was traced back to WHO IS as 629 IU/ml based on the results of pseudo virus neutralization assay in lab ZX. These national standards have been used in the detection and evaluation of neutralizing antibodies of products against COVID-19 in China, and will greatly promote the development of preventive vaccines and therapeutic antibodies for COVID-19.

## 6 Limitations of the study

The collaborative study of the first Chinese national standard for SARS-CoV-2 neutralizing antibody only used the methods of detecting the neutralizing antibody titer, and did not include the methods of detecting the binding antibody such as ELISA. The neutralization potency of the first Chinese national standard for SARS-CoV-2 neutralizing antibody was affected by spike substitutions found in variants of concern (VOC). Compared with the Wuhan-1 reference strain (Wild Type), the neutralizing titer of the national standard against B.1.1.7 decreased by 1.9-fold, and the neutralizing titer against B.1.351 and P.1 reduced by more than 10-fold (Figure S1). In the future, we will establish the second-generation Chinese national standards for SARS-CoV-2 neutralizing antibody that can effectively neutralize VOC.

## Declaration of Competing Interest

The authors declare that they have no known competing financial interests or personal relationships that could have appeared to influence the work reported in this paper.
