# Supplementary material for: The first Chinese national standards for SARS-CoV-2 neutralizing antibody
Source: Vaccine. 2021 Jun 23;39(28):3724–30. doi: 10.1016/j.vaccine.2021.05.047 (PMC8133612; doi:10.1016/j.vaccine.2021.05.047)
Supplement: Supplementary data 1 [file mmc1.docx]

**Table S1. The neutralizing titer for samples in the pseudo virus neutralization assay**

| **Lab** | | **Assay** | **Sample** | | | | | | | | | | |
| --- | --- | --- | --- | --- | --- | --- | --- | --- | --- | --- | --- | --- | --- |
|  |  |  | ***22*** | | | ***44*** | ***55*** | | | ***77*** | ***99*** | |  |
| WS1 | 1 | | | 1886 | 3857 | | | ＜30 | 125 | | | 1679 | |
|  | 2 | | | 1657 | 4214 | | | ＜30 | 114 | | | 1892 | |
|  | 3 | | | 1959 | 5159 | | | ＜30 | 183 | | | 1791 | |
|  | 4 | | | 1040 | 4452 | | | ＜30 | 107 | | | 1675 | |
|  | 5 | | | 1635 | 3941 | | | ＜30 | 140 | | | 1387 | |
|  | 6 | | | 1651 | 5166 | | | ＜30 | 137 | | | 1786 | |
|  | 7 | | | 5135 | ＞7290 | | | ＜30 | 111 | | | 1315 | |
|  | 8 | | | 1710 | 3505 | | | ＜30 | 56 | | | 1699 | |
|  | 9 | | | 1560 | 4435 | | | ＜30 | 91 | | | 2388 | |
| ZX | 1 | | | 1542 | 2590 | | | ＜30 | 123 | | | 1620 | |
|  | 2 | | | 1803 | 2355 | | | ＜30 | 94 | | | 1374 | |
|  | 3 | | | 1349 | 2846 | | | ＜30 | 131 | | | 1485 | |
|  | 4 | | | 1213 | 2220 | | | ＜30 | 71 | | | 1429 | |
| SZ | 1 | | | 1429 | 3998 | | | ＜30 | 42 | | | 4542 | |
|  | 2 | | | 1575 | 2540 | | | ＜30 | 102 | | | 2055 | |
|  | 3 | | | 1369 | 2780 | | | ＜30 | 126 | | | 2830 | |
| ZA | 1 | | | 4186 | >7290 | | | ＜30 | 184 | | | 4166 | |
|  | 2 | | | 2108 | 5076 | | | ＜30 | 146 | | | 3130 | |
|  | 3 | | | 3966 | >7290 | | | ＜30 | 160 | | | 3314 | |
| ZC | 1 | | | 1873 | 3521 | | | ＜30 | 96 | | | 2329 | |
|  | 2 | | | 1534 | 4249 | | | ＜30 | 73 | | | 4227 | |
|  | 3 | | | 1169 | 3496 | | | ＜30 | 38 | | | 1210 | |
| ZW | 1 | | | 4182 | 11603 | | | ＜50 | 392 | | | 5919 | |
|  | 2 | | | 8586 | ＞12150 | | | ＜50 | 6876 | | | 9575 | |
|  | 3 | | | 6374 | ＞12150 | | | ＜50 | 1197 | | | ＞12150 | |
| JW | 1 | | | 1569 | 5584 | | | ＜30 | 124 | | | 1812 | |
|  | 2 | | | ＞2430 | 2322 | | | ＜30 | 76 | | | 1594 | |
|  | 3 | | | 1787 | 3149 | | | ＜30 | 78 | | | 2942 | |
|  | 4 | | | 3720 | ＞7290 | | | ＜30 | 77 | | | 1421 | |
| JS1 | 1 | | | 1383 | 2614 | | | ＜30 | 112 | | | 1543 | |
|  | 2 | | | 1223 | 2903 | | | ＜30 | 131 | | | 2195 | |
|  | 3 | | | 1424 | 3521 | | | ＜30 | 142 | | | 1539 | |
|  | 4 | | | 1075 | 2804 | | | ＜30 | 124 | | | 1651 | |
| BS | 1 | | | 1756 | 2736 | | | ＜30 | 170 | | | 918 | |
|  | 2 | | | 1352 | 3037 | | | ＜30 | 124 | | | 777 | |
|  | 3 | | | 1235 | 2495 | | | ＜30 | 82 | | | 518 | |
| ZY1 | 1 | | | 3107 | 4224 | | | ＜30 | 1264 | | | 2219 | |
|  | 2 | | | 1401 | 6435 | | | ＜30 | 380 | | | 1110 | |
|  | 3 | | | 1420 | 4322 | | | ＜30 | 540 | | | 1002 | |

**Table S2. The neutralizing titer for samples in the live virus neutralization assay**

| **Lab** | | **Assay** | | **Sample** | | | | | | | | | |
| --- | --- | --- | --- | --- | --- | --- | --- | --- | --- | --- | --- | --- | --- |
|  |  |  |  | ***22*** | ***44*** | | | ***55*** | | ***77*** | ***99*** | |  |
| BK | 1 | | 768 | | | 1536 | ＜8 | | 128 | | | 1536 | |
|  | 2 | | 1536 | | | 1536 | ＜8 | | 256 | | | 1536 | |
|  | 3 | | 1536 | | | 2048 | ＜8 | | 128 | | | 1536 | |
| JS2 | 1 | | 45 | | | 385 | ＜16 | | ＜16 | | | 260 | |
|  | 2 | | 20 | | | 260 | ＜16 | | ＜16 | | | 245 | |
|  | 3 | | 53 | | | 297 | ＜16 | | ＜16 | | | 258 | |
|  | 4 | | 27 | | | 251 | ＜16 | | ＜16 | | | 114 | |
|  | 5 | | 29 | | | 239 | ＜16 | | ＜16 | | | 174 | |
| ZY2 | 1 | | 192 | | | 1024 | ＜8 | | 24 | | | 512 | |
|  | 2 | | 96 | | | 512 | ＜8 | | 8 | | | 384 | |
|  | 3 | | 96 | | | 512 | ＜8 | | 16 | | | 256 | |
| WS2 | 1 | | 1094 | | | 2535 | ＜30 | | 326 | | | 1748 | |
|  | 2 | | 932 | | | 2031 | ＜30 | | 209 | | | 1787 | |
|  | 3 | | 1179 | | | 2388 | ＜30 | | 90 | | | 1004 | |
| WS3 | 1 | | 144 | | | 1152 | ＜12 | | 18 | | | 288 | |
|  | 2 | | 144 | | | 1152 | ＜12 | | 36 | | | 768 | |
|  | 3 | | 288 | | | 1152 | ＜12 | | 18 | | | 576 | |
|  | 4 | | 288 | | | 1152 | ＜12 | | 18 | | | 576 | |
|  | 5 | | 288 | | | 1152 | ＜12 | | 36 | | | 192 | |
|  | 6 | | 144 | | | 1152 | ＜12 | | 36 | | | 576 | |
|  | 7 | | 192 | | | 576 | ＜12 | | 24 | | | 384 | |
|  | 8 | | 192 | | | 576 | ＜12 | | 18 | | | 384 | |
|  | 9 | | 192 | | | 576 | ＜12 | | 24 | | | 384 | |


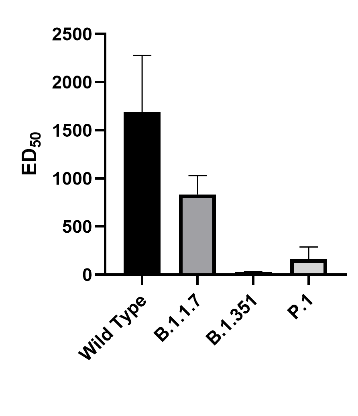


Figure S1. Neutralization activity of the first Chinese national standard against variants of concern. ED50, median effective dilution.
